# Development of a Plug-and-Play Monitoring System for Cabled Observatories in the East China Sea

**DOI:** 10.3390/s150817926

**Published:** 2015-07-23

**Authors:** Yang Yu, Huiping Xu, Changwei Xu

**Affiliations:** State Key Laboratory of Marine Geology, Tongji University, 1239 Siping Road, Shanghai 200092, China; E-Mails: 10yuyang@tongji.edu.cn (Y.Y.); xcw@tongji.edu.cn (C.X.)

**Keywords:** cabled observatories, ocean sensor network, remote control, monitoring system, plug-n-play

## Abstract

Seafloor observatories enable long term, continuous and multidisciplinary observations, promising major breakthroughs in marine environment research. The effort to remotely control *in situ* multidisciplinary equipment performing individual and cooperative tasks is both a challenge and a guarantee for the stable operations of functional observatories. With China starting to establish ocean observatory sensor networks, in this study we describe a monitoring system for cabled observatories in the East China Sea (ESOMS) that enables this effort in a plug and play way. An information oriented monitoring architecture for ESOMS was first introduced, derived from a layered control model for ocean observatory sensor network. The architecture contained three components and enabled bidirectional information flow of observation data and commands, based on which architecture components were designed to enable plug-and-play control within related model layers. A control method enabled by general junction box (GJB) and ocean sensor markup language (OSML) was thus proposed as the plug-and-play solution for implementing ESOMS. The GJB-OSML enabled control method (GOE Control Method) mainly actualized two processes, one of which was that the *in situ* GJB interfaced and represented every attached sensor as a Sensing Endpoint in the cabled observatory network. The other process was that the remote ESOMS utilized the same IP/Port related information modeled by OSML to create/operate a Function Node acted as agent of the *in situ* sensor. A case study for using ESOMS in the Xiaoqushan Seafloor Observatory was finally presented to prove its performance and applicability. Given this successful engineering trial, the ESOMS design and implementation could be applicable and beneficial for similar efforts in future construction of seafloor observatory network both at home and abroad.

## 1. Introduction

Research over the past 20 years has strongly suggested the key role oceans play in global climate change, with a high demand for long-term and continuous observation data [1]. Emerging in response to this demand, seafloor observatories have become a key component of international marine research and are widely recognized as the third observation platform for geosciences following remote sensing [2,3]. The original idea of deploying seafloor observatories comes from the U.S. Navy’s Sound Surveillance System (SOSUS) facilitated during the Cold War [4], designed to put a set of multifunctional observation equipment under the sea and use wired or wireless networks to supply power and collect information. In this way, seafloor observatories enable long-term, continuous, real-time, weather independent and multidisciplinary scientific observations [5]. MARS (Monterey Accelerated Research System) and VENUS (Victoria Experimental Network Under the Sea) represent operational test beds for deep-sea observatory instruments [6], while NEPTUNE (North-East Pacific Time-series Undersea Networked Experiments) is the largest cabled observatory established so far [7]. Some advanced projects under construction include the OOI (Ocean Observatories Initiative) funded by the National Science Foundation of the USA [8], the EMSO (the European Multidisciplinary Seafloor Observatory) in Europe [9] and Ocean Network Canada Observatory.

Three prerequisites exist for constructing a cabled seafloor observatory network. Firstly, observation sites must be located in deep sea environments to ensure long-term and continuous observation. Secondly, power supply and information transmission must be networked. Thirdly, junction boxes are commonly used to guarantee power conversion and supply as well as automatic recoding and transmission of observation data [10]. Once established, a seafloor observatory network is expected to contribute to basic scientific research, resources exploration and energy exploitation, disaster prevention and environment protection, military application and so on [11]. Continuous measurements in physics, chemistry, biology and geology are made within the whole water body. These multidisciplinary data sets will resolve scientific processes in a broad range of temporal scales and particularly promote researches on ocean’s role in climate change, fluids and life in the oceanic crust, dynamics of oceanic lithosphere and imaging earth’s interior, turbulent mixing and biophysical interaction, ecosystem dynamics and biodiversity, and coastal ocean processes [12].

The East China Sea (ECS) has increasingly attracted attentions from both scientific communities and the public over the past decade owing to the huge fluvial discharges from mainland China and Taiwan Island, the intense anthropogenic activities in estuarine and coastal areas, and the remarkable environmental changes at present and during the Quaternary [1]. The ECS has a broad shallow shelf and can therefore be regarded as an ideal site for studying a source to sink system, land-sea and natural-anthropogenic interactions on a broad range of spatial and temporal scales. Accordingly, the Xiaoqushan Seafloor Observatory [13] in the ECS was first built with support from the Science and Technology Commission of Shanghai in 2006 aiming at “Testing and Preliminary Application of Seafloor Observatory Networking Technologies”. As the first cabled observatory in China, the Xiaoqushan Seafloor Observatory was upgraded in 2013 to serve as the first test bed for a new long-term instrumentation and an integrated observation station [14]. This successful observatory lays a solid foundation for accumulating networking technologies under the sea, and contributes to the plan for more integrated and advanced cabled observatories in the ECS. In order to guarantee the stable operations of these cabled observatories, the demands of monitoring and controlling *in situ* multidisciplinary equipment to perform individual and cooperative tasks must be fully met. It is upon this scientific and engineering demand that a preliminary study has been carried out to design and develop a monitoring system for cabled observatories in the East China Sea (ESOMS).

The paper is organized as follows. In Section 2, we introduce the information oriented monitoring architecture for ESOMS. In Section 3, we describe the plug and play implementation of ESOMS. In Section 4, we do a case study for using ESOMS in the Xiaoqushan Seafloor Observatory and provide an evaluation on its practical performance. In Section 5, we discuss the contributions and provide conclusions.

## 2. Architecture Design for ESOMS

### 2.1. Control Model Derived Architecture

A control model for an ocean observatory sensor network was proposed to design the monitoring architecture for ESOMS. This model was information oriented and was structured into four layers that contributed to vertical and horizontal integration of the observatory sensor network. The sensing layer sensed dynamic marine environment, forming the information base for the control model. The collecting layer collected *in situ* observation data and relayed information between the service layer and the sensing layer. The service layer managed various types of information and provided a set of operations related to remote control. The application layer was an engineering oriented layer that used different information to meet requirements laid for the project. Layers were relatively functionally independent with interfaces between them defined in a standard way. Each layer used services from its adjacent bottom layer and provided services to its adjacent upper layer. Layers can be deployed flexibly along the path where the bidirectional information flow was transmitted, and the monitoring architecture we designed for ESOMS was just one of the deployment scenarios derived from this model.

**Figure 1:**
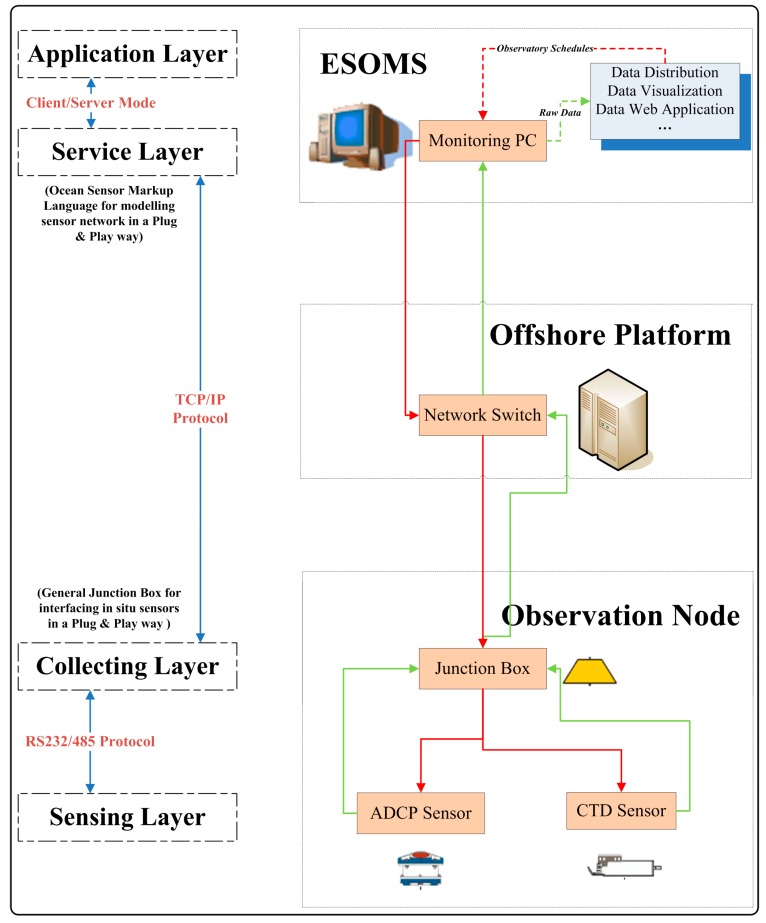
Information Oriented Architecture for ESOMS.

As shown in Figure 1, this information oriented architecture for ESOMS contained three components (Observation Node, Offshore Platform, and ESOMS) and two types of information flow (flow of data collected and flow of commands). The flow of data (green) contains *in situ* observation data gathered from cabled observatories in the ECS; while the flow of commands (red) stands for types of commands sent to control undersea sensors within the Observation Node in accordance with real-time monitoring schedules.

The architecture related to the layered control model in the following way. The application layer and the service layer were deployed within ESOMS while the collecting layer and the sensing layer were distributed within the Observation Node. An ocean sensor markup language was designed within the service layer to model sensor information in a standardized way, which contributed to managing and controlling the sensor network in a plug and play mode at the software level. The general junction box designed within the collecting layer can interface and network *in situ* sensors in a plug and play mode at the hardware level [15], thus enabling a system level of plug and play mode for newly added or reconfigured ocean sensors within the Observation Node.

### 2.2. Operational Information Flow

The *in situ* junction box, one ADCP sensor, and one CTD sensor, constituted the Observation Node of cabled observatories. Observation data captured by deployed sensors were first transmitted to the communication control module of the junction box for encoding, and then forwarded to the network switch on the offshore platform for buffering via the submarine electro-optic cable under TCP/IP protocol. Upon arriving at the switch, all collected data entered the remote transmission phase and were received and stored by ESOMS running at the control center in a real-time way (as the green information flow in Figure 1). All the received raw data were stored in the database and simultaneously made backup in notepad (.txt) files in consideration of the system’s current demands. In order to complete the data processing chain, a data distribution system was designed to fulfill data acquisition and transmission, data interpretation and storage, and data visualization. Furthermore, a web application system was implemented to enable data access over the Internet. Data access was only permitted to registered researchers in this prototype system, which provided services such as map browsing, real-time data query, time-series data curve, historical data chart, and data download. 

ESOMS was, on the one hand, responsible for receiving real-time data packets and preparing them for further data processing or application. On the other hand, this system was mainly in charge of remotely monitoring and controlling various types of observation equipment under the sea, according to real-time and dynamic observation schedules. Commands were reversely transmitted in the same communication channel and under the same communication protocols as how data were transmitted to ESOMS. The junction box played the role in assigning these commands to specific underwater sensors to control their working status (as the red information flow in Figure 1).

## 3. Plug-and-Play Implementation of ESOMS

### 3.1. Plug-and-Play Solution

We defined plug and play as a process that fulfills the standardization of sensor communication and control in cabled observatories. In this process, any newly added sensor was dynamically networked under the sea and simultaneously remotely controlled in a hot swapping way, without interfering current sensors’ control operations. In the context of ESOMS monitoring architecture (Figure 1), the process of plug and play was respectively enabled in the collecting layer and the service layer. The general junction box (GJB) introduced in the collecting layer could dynamically interface and network attached marine sensors; while the ocean sensor markup language (OSML) implemented in the service layer could model sensor information in a standardized way and contribute to the remote hot swapping control. We thus proposed a GJB-OSML enabled control method (GOE Control Method) as the plug-and-play solution for implementing ESOMS, based on which the remote client in the application layer could instantly control any sensor newly added in the sensing layer.

With the monitoring architecture and GOE Control Method proposed, ESOMS was designed as a web-based, module-integrated monitoring system [16]. In terms of hardware communication (Figure 1), the decision was taken that communication between the monitoring PC and the junction box complied with TCP/IP protocol [17,18] while RS232/485 serial communication was used between the junction box and various sensors attached [19]. As for software design, remote monitoring programs using socket technologies are usually divided into client and server components [20]. Based on the configuration principle that modules should be mixed randomly [21], a communication control module was introduced in the general junction box to act as the server component for listening, connecting, receiving and sending data. In this case, ESOMS was implemented as a client program structured into five modules, namely, a remote monitoring module, an information retrieval module, an information management module, a system management module and a user management module.

Real-time remote monitoring was thus enabled in the following process. First, each sensor was addressed and a socket connection was established using the unique pair of IP address and port number. Data transmission was then performed using the established socket connection, and all the *in situ* data and messages related to remote monitoring were synchronously written into the formatted log files and data files. Meanwhile, various commands were dynamically sent to reconfigure operating parameters of undersea observation equipment, using the same socket connection.

### 3.2. GOE Control Method

**Figure 2:**
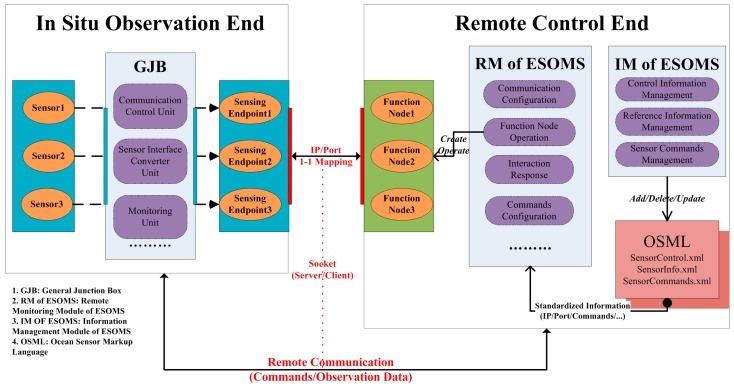
GOE Control Method.

The GOE Control Method was generally implemented in the following way. Firstly, the *in situ* GJB interfaced and represented every attached sensor as a Sensing Endpoint in the cabled observatory network. A Sensing Endpoint was identified by a unique pair of IP address and port number, and thus can be addressed and remotely communicated in bidirectional information flow. Secondly, the remote ESOMS utilized the same IP/Port related information modeled by OSML to create/operate a Function Node that acted as agent of the *in situ* sensor. In this way, 1-1 mapping was made between every Sensing Endpoint and every Function Node by IP/port number pair, which was further passed to socket as parameters to establish remote communication. Control commands can be finally transmitted via this remote communication for configuring sensors and getting observation data (Figure 2).

GOE Control Method laid the foundation for the standardization of sensor communication and control at the system level. For any newly added sensor in the sensing layer, the GJB in the collecting layer would interface the sensor and represent it as a network resource of Sensing Endpoint in an IP-based standardized way. Meanwhile, in the service layer, ESOMS would utilize OSML standardized information to create/operate the mapping Function Node without interfering currently operational sensors or other system operations. In this way, the application layer demanded no additional design for new sensors in the sensing layer and was thus enabled a plug-and-play solution for the bidirectional transmission of observation data/commands.

In the following sub sections, GOE Control Method was detailed in three parts explaining principles of the method.

#### 3.2.1. GJB for Interfacing and Networking Sensors

As shown in Figure 2, the general junction box (GJB) was integrated with a communication control unit, a sensor interface converter unit, a monitoring unit, a photoelectric signal conversion unit, a power supply and allocation unit, and so on. Regarding the standardization of sensor communication and control via GJB, the communication control unit played the most important role in completing the transparent protocol conversion from serial interfaces to network transmission. To be specific, this unit contained a smart communication server that used TCP/IP protocol and worked in a server socket mode. The server was given a standalone IP address and corresponded every physical port to a unique port number. Thus, every ocean sensor attached to GJB was automatically allocated a unique pair of IP address/port number and was represented as a Sensing Endpoint in the network. Once a socket connection was established with a unique pair of IP/port information offered by a Sensing Endpoint, the sensor attached to that corresponding physical port was exposed on the Internet and the interactive transmission of observation data/control commands can be performed using this connection. 

Based on the communication control unit and other functional units, GJB succeeded in interfacing ocean sensors in cabled observatories and networking them as corresponding Sensing Endpoints ready for bidirectional remote communications. This contributed to standardizing sensor communication and control in the sensing layer and the collecting layer.

#### 3.2.2. OSML for Modeling and Controlling Sensors

Extensible Markup Language (XML) is a markup language that defines a set of rules for encoding documents in a format which is both human-readable and machine-readable. XML data is known as self-describing, the basic building block of which is an element defined by tags. Many application programming interfaces (APIs) have been developed to aid the processing of XML data, while applications for the Microsoft .NET Framework commonly use XML files for system configuration. Based on XML and the Microsoft .NET Framework, the Ocean Sensor Markup Language (OSML) was designed and developed as a common information model contributing to remotely controlling and managing the cabled observatory sensor network in a plug-and-play way.

As there was no limitation to a set of tags, OSML was designed with customized elements that both precisely defined functional requirements laid for ESOMS and described various types of information for controlling/managing *in situ* sensors. Data access for a selected sensor’s control and management information was accomplished using OSML and was operated via VB.NET code. Two major instantiated OSML files were SensorControl.xml and SensorInfo.xml, the former of which mainly related to establishing a remote connection while the latter of which included supplement information for undersea equipment. The management of a sensor’s command information mainly utilized component technology [22], which was programmed to display and manage various types of control commands in a tree structure in the main interface using methods provided by the dynamic link library and .NET class, and corresponding instantiated OSML files.

With OSML properly marked up and its elements properly nested, component-oriented modules of ESOMS can process the OSML tags in a standardized and dynamic way as follows (Figure 2). Once any current sensor needed reconfiguration or any new sensor was added, Information Management Module called corresponding methods to update SensorControl.xml and SensorInfo.xml without affecting current operations of the whole system. Remote Monitoring Module of ESOMS then loaded the updated information such as IP address/port number, established remote connections, and performed control operations on re-configure or newly added sensors without interfering with other *in situ* equipment. This design enabled a better remote control and management of cabled observatory sensor network in the ECS.

#### 3.2.3. Function Node for Remote Communication

Network process communication lays the foundation for remote monitoring. In terms of ESOMS, a client-server architecture based on the .NET Framework Socket class [23,24] was adopted to address the issue of remote data communication. The TCP/IP protocol was chosen as the main communication protocol for the network application, in which the concept of IP address and port number was introduced to uniquely identify the communication process. Once a socket connection was established, remote communication was enabled and the interactive transmission of both data and commands were performed using this connection.

**Figure 3:**
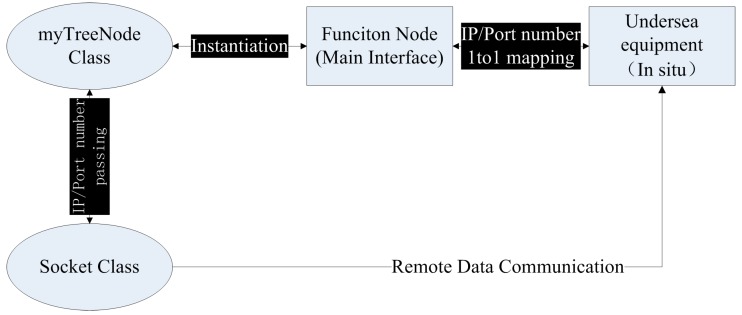
Function Node Design for ESOMS.

Since a one-to-one correspondence existed between every sensor in the cabled observatories and a unique IP address and port number pair, a mapping was established by creating a “Function Node” in the Main Interface that acted as agent of the *in situ* observation device. This process was introduced in detail as follows. A “myTreeNode” class encapsulating the IP address and port number as new public members was firstly inherited from the TreeNode class of .NET Class Library. The myTreeNode object then used the IP address and port number as parameters when a Socket object was initialized or a socket connection was established. Thus, the one-to-one binding between a myTreeNode object and a Socket object can be performed, and creating a Function Node in the Main Interface was made equal to adding a myTreeNode object to the Treeview Control of the Main Interface. By using these object-oriented programming techniques, the binding connection between Socket objects and *in situ* observation equipment was successfully established which addressed the remote communication challenges of ESOMS (seen in Figure 3). Combining with OSML, this Function Node design enabled the remote communication and interaction with every ocean sensor in a plug and play mode.

### 3.3. System Optimization

In the construction of cabled observatories, especially for developing ESOMS, a number of technological challenges should be taken into account to guarantee the stable operations of the whole system. In order to monitor the real-time status of each Function Node and to remotely control the corresponding *in situ* Sensing Endpoint, some additional designs were implemented and highlighted as follows.

#### 3.3.1. Communication Optimization

From an operational point of view, a cabled observatory network consisted of a communication system and a power system. Both must be monitored and controlled in a real-time way. As for the communication side, we firstly developed an automatic connection algorithm to determine and control the network connection status. By using multi-thread processes, Timer class, and Socket class in blocking mode, an testing string was regularly send to all the Sensing Endpoints at the remote general junction box to assess the connection status of all the Function Nodes. If a node was disconnected accidentally but still demanded real-time remote control, ESOMS could automatically re-establish the socket connection and re-transmit both control commands and observation data.

In addition, we implemented the concept of Status Indicator to monitor the working status of all the *in situ* sensors. As for each Function Node added to the Operating Area of the Main Interface, TreeView Control and Imagelist Control were programmed to display and indicate its current status by means of three colors of Indicator. To be specific, conventions were used that a Red Indicator stood for not connected or disconnected while a Yellow Indicator stood for connected only. A Green Indicator stood for connected as well as in use. An algorithm was written to determine the current status of all nodes and to change the color of the Indicator in the display.

As every operation was logged in the log file, log file data were accessed in the following way: every day the running system automatically created a log file named the date of that day in the Log Folder and synchronously wrote all the remote control operations performed in the system into that log file with .txt format.

#### 3.3.2. Power System Optimization

On the power side, a power monitoring and control system (PMACS) was implemented to monitor and control overall electric energy of cabled observatories [25]. PMACS consisted of a data acquisition system (DAS), a load management system (LMS) and a network analysis system (NAS). Based on state estimation and fault location, PMACS combined status data with mathematical model to estimate, predict and provide strategies to the operations of power system. PMACS also controlled the power supply to all the internal and external loads, which further contributed to the improvement of energy efficiency and operation reliability.

As the submarine cables were of considerable length, power loss over transmission distance must be taken into consideration. If the maximum output voltage of shore-based power feeding equipment (PFE) was U_SS_, the normal input voltage for undersea converters must be between 0.5 U_SS_ and U_SS_ to ensure the stability of power system. LMS was thus designed with a load management strategy to guarantee that the input voltage of all the undersea stations met this restriction. Cable fault was also a technological challenge closely related to the stability of power system, the main form of which was ground fault. When a ground fault happened, PMACS adjusted PFE to low voltage and stopped all the undersea stations for the safety of the whole cabled observatory network. The fault location module of NAS was applied to work out the cable section of ground fault and reconstruct the power network topology, thus realizing ground fault location.

*In situ* sensors were ensured correct power intensity by PMACS in the following way. A PFE in the shore station firstly converted the AC electric power to −2 KV DC electric power, which was delivered to undersea stations via the submarine cable and branching unit (BU). Undersea stations then converted the high-voltage electric power to medium-voltage (MV) electric power and supplied the MV power to general junction boxes (GJBs) by the extension cable. Finally, GJB converted the 375 V DC electric power to 48/24/12 V DC electric power and fed them to multiple science loads via instrument cables.

#### 3.3.3. Centralized Management for *in Situ* Sensors

**Figure 4:**
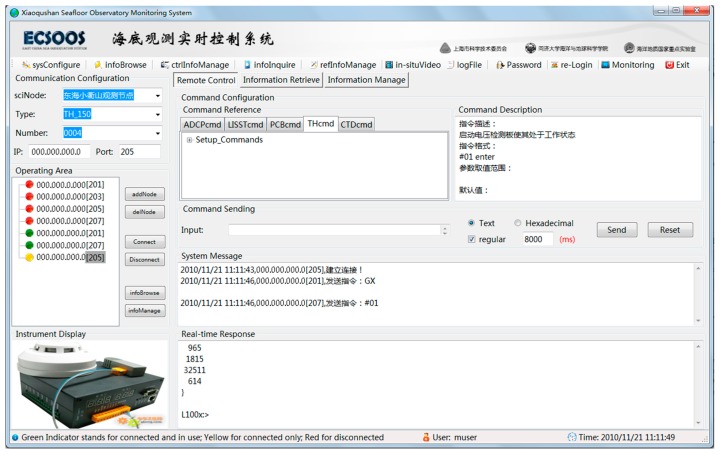
Main Interface of ESOMS.

Sensors were located within different Observation Nodes and thus demanded a centralized design for distributed control of all the *in situ* sensors. We implemented the centralized management design at the remote control end by combining the customized myTreeNode class with TreeView Control object of .NET. This not only loaded the control information (IP address and port number) from database to the Main Interface and displayed them in the Operating Area, but also performed a centralized management of all the Function Nodes (corresponding to every *in situ* sensor) currently in use (Figure 4). In addition, some public variables were set to put together all the function buttons for remotely controlling Function Nodes in the Operating Area and Command Sending Area. Once a Function Node was selected, all the current control operations were executed only for this chosen one until other Function Nodes were selected. Besides, the Command Reference Area and Instrument Display Area would respectively navigate to the command content and in-kind image of the sensor corresponding to the chosen Function Node in the Main Interface.

## 4. A Case Study for Using ESOMS

In this section, a brief case study describing the use of ESOMS in the Xiaoqushan Seafloor Observatory was presented. Experiment scenarios were firstly introduced prior to experiment processes and results. System performance and initial outcome were then given in support of the ESOMS applicability.

### 4.1. Experimental Scenario: Xiaoqushan Seafloor Observatory

The inner shelf off the Yangtze estuary is of particular interest, as strong seasonal variability of the Yangtze River-derived nutrient inputs and marine productivity are closely related to the occurrence of upwelling, red tide and hypoxia [26]. The focused area is roughly located in the inner shelf off Shanghai where different waters originating from the Yangtze River and the Open Ocean are confluent, and thus configure strong land-sea interactions. This focused area has also been subject to rapidly increasing anthropogenic impacts such as harbor engineering and fishing activities over the past two decades. The Xiaoqushan Seafloor Observatory is established precisely within this focused area, geographically between 30°31′44′′ N, 122°15′12′′ E and 30°31′34′′ N, 122°14′40′′ E.

As a coastal observatory, the Xiaoqushan Seafloor Observatory originally consisted of a double-armored composite optical cable that was 1.1 km long, a special junction box, an ADCP (Acoustic Doppler Current Profiler) sensor, a CTD (Conductivity Temperature Depth) sensor, an OBS (Optical Back Scattering) sensor and an anti-trawl frame. The special junction box was integrated with a power control module, a communication control module, an interface module, a protection module and a photovoltaic conversion module. The deployed sensors were connected to this junction box via standard watertight plug interfaces. The cable landing site was made at the offshore platform of the East China Sea Branch of State Oceanic Administration, where a solar battery was offering long-term and continuous power. The data collected from various instruments were firstly encoded by the junction box and transmitted to the offshore platform through the fiber optic cable, and then forwarded to the data center at the State Key Laboratory of Marine Geology, Tongji University, via CDMA (Code Division Multiple Access) wireless transmission [13].

Observatory upgrades began in October 2011 and ended in August 2013, transforming it into an integrated observation station and a test bed for oceanographic instrumentation. The upgrades mainly covered three aspects. The first upgrade was the general junction box designed to enable the plug and play interfacing for various types of ocean sensors. This new junction box had one optical fiber plug for connecting the backbone cable and over 10 universal waterproof plugs for sensors, making the observatory a real test bed for scientific instrumentation. The second upgrade was a series of reconstruction work. A few more solar panels were added to the top of the platform’s edge and a windmill was installed, while the platform’s communication and remote control equipment were also upgraded to handle the increasing amount of transmitted data. The third upgrade was that more sensors for multidisciplinary observation were integrated into the observatory (Table 1), including sensors for physical oceanography and marine geochemistry, as well as geological and geophysical equipment. A CO_2_ sensor was installed on the top of the offshore platform to measure sea-surface atmospheric CO_2_ concentration for comparison with seafloor observation sensor data.

**Table 1:** Sensor Upgrade for Xiaoqushan Seafloor Observatory.

Sensor	Original Observatory	Upgrade Observatory
Video	√	√
CO_2_	×	√
OBS	×	√
CTD	√	√
Turbidity	√	√
Underwater CO_2_	×	√
PH	×	√
Tide and Wave	×	√
ADCP	√	√
Imaging Sonar	×	√
Magnetometer	×	√

### 4.2. Prototype System Test: Processes and Results

Based on the information oriented architecture and GOE control method, ESOMS was implemented as a module-integrated monitoring system and was tested for its plug-and-play control through a series of tests. ESOMS was first performed a tank test in the Seafloor Observatory Laboratory at Tongji University. During the testing processes a general junction box and several types of ocean sensors were simulated as the *in situ* observatory and were continuously debugged under both normal operations and boundary conditions. The tank test proved that ESOMS was capable of dynamically controlling remote sensors in a plug-and-play way and maintaining an uninterrupted operation for as long as 2000 h. The whole system was then successfully tested for a shallow sea trial before it was finally put into service for the Xiaoqushan Seafloor Observatory.

Continuous data sets and various observatory operations demonstrated strong demands for using ESOMS in the Xiaoqushan Seafloor Observatory, with remote monitoring and control being the most important operational process. This process was mainly executed by the remote monitoring module of ESOMS, which was divided into several sub modules respectively responsible for communication configuration, command configuration, function node operation, interaction response, and real-time monitoring. The module interface was the Main Interface of ESOMS containing five function areas in accordance with the five functional sub modules as follows: (i) Communication Configuration Area: configured the IP address and port number for the *in situ* sensor with specified information of junction box node, sensor type and sensor number for remote control; (ii) Command Configuration Area: loaded and displayed the commands for remotely controlling *in situ* sensors, configured and sent the control commands in accordance with real-time demands; (iii) Operating Area: displayed and managed all the Function Nodes in the centralized form of list tree, executed basic remote control operations such as remote connection/disconnection and performed the command control/information browsing/information management for the selected Function Node; (iv) System Message Area: displayed the entire real-time system message related to remote connection/disconnection and control commands sending; (v) Real-time Response Area: displayed the real-time received data and system message. The test result for remote monitoring and control process was shown in the Main Interface of ESOMS (Figure 4).

**Figure 5:**
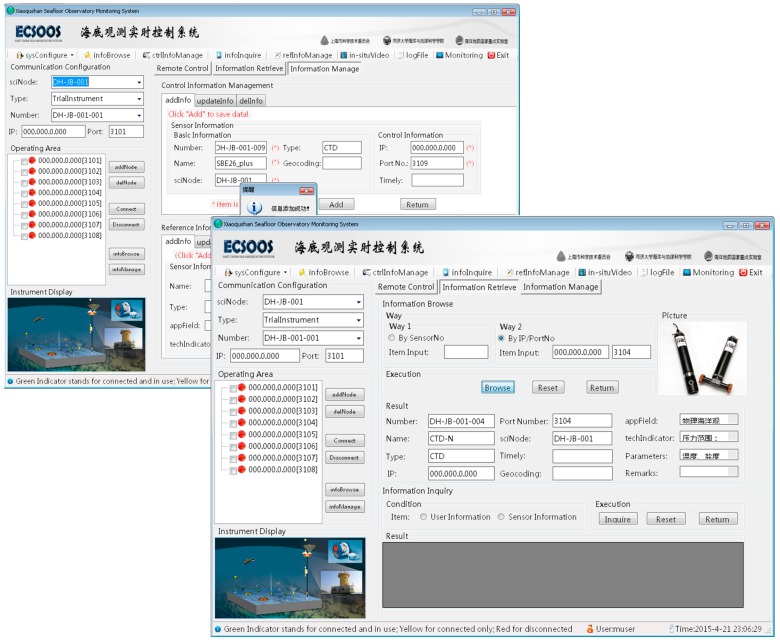
Rendering of the Information Management and Retrieval for ESOMS.

All the five modules of ESOMS were well implemented with functionality described in the next paragraph. As stated above that the remote monitoring module was mainly responsible for the remote monitoring and control process, the information management module took on the process of managing sensor control information modeled by OSML and updating the information in both foreground interfaces and background database (Figure 2). Other modules functioned and contributed to a dynamic, robust, and complete monitoring system for cabled observatories.

(a) The remote monitoring module of ESOMS was implemented to dynamically receive and store all the *in situ* data, on-line monitor the Xiaoqushan Seafloor Observatory, autonomously interpret raw data for event detection and response, and remotely control undersea observation equipment.
(b) The information management module of ESOMS was implemented to dynamically manage (add, delete or edit) and refresh these three types of sensor information (seen in Figure 5) for remote monitoring in the Main Interface, and synchronously update the changes in the database.
(c) The information retrieval module of ESOMS was implemented (seen in Figure 5) to enable operators to know all the information related to deployed sensors in the cabled observatory, and provide statistical information distribution.
(d) The system management module of ESOMS was implemented to configure the refresh rate for data display containers like System Message Area and Real-time Response Area while executing remote control operations, and to query and browse historical operations in ESOMS.
(e) The user management module of ESOMS was implemented to enhance the system security through 3 layers filtering: (i) invalidated users were not allowed to use the monitoring system; (ii) every registered user was assigned different levels of management authority to perform operations in ESOMS (gray menu in the Main Interface was currently unavailable); (iii) ESOMS automatically recorded any operation any user performed in the log file, defining responsibility to some extent.

### 4.3. System Performance and Initial Outcome

Since ESOMS was put into service for the Xiaoqushan Seafloor Observatory, it has been consistently functioning and presenting satisfactory practical performance in accordance with all the requirements laid for the project:

(1) All data collected from the Xiaoqushan Seafloor Observatory were received, interpreted and stored in a real-time way. Science data were reliably associated with metadata and performed a data quality control.
(2) All facilities within the Xiaoqushan Seafloor Observatory and various marine environment parameters were on-line monitored, and the deployed sensors were controllable from shore-based platforms. Operators on shore can interactively modify data acquisition schedules and other working parameters for the observatory sensor network, and can remotely diagnose the cabled observatory.
(3) ESOMS was able to autonomously detect events of interest and quickly respond to them. Some events of interest may occur when operators were not actively examining the data stream for events (e.g., late at night), and thus demanded that ESOMS can automatically detect these events in the instrument data streams and respond with predefined remote control capabilities. This feature enabled adaptive *in situ* experiments and obtained more scientific outcomes.
(4) The Xiaoqushan Seafloor Observatory was able to support oceanographic instrumentation for shallow water trial, including commercial sensors and customized ones. The process of adding/removing observation nodes and sensors from the deployed system was simple, robust and scalable to the cabled observatory when demands emerged for carrying out more experimental tasks under sea.
(5) The ESOMS architecture enabled interaction with other kinds of devices and systems, such as autonomous underwater vehicles and different kinds of moorings or cable-shore systems.

Given the hostility of the marine environment in the ECS to underwater observation equipment, the remote control capabilities of ESOMS guaranteed that the Xiaoqushan Seafloor Observatory could provide a long, continuous and adaptive data record for studying sea-land interactions in the region. It may be too early to judge the output from the Xiaoqushan Seafloor Observatory even only in the regional oceanic context. However, the available operational period of ESOMS functioned on the Xiaoqushan Seafloor Observatory showed very clearly a record of considerable variability on time scales that were not captured by a routine boat survey. An initial analysis of some *in situ* observation data sets from the Xiaoqushan Seafloor Observatory [13] demonstrated that the long-term measurements contributed to comprehensive studies on the mechanisms of ocean dynamics and environmental variations under different weather conditions. Another initial outcome was the observatory data record and analysis of the tsunami induced by the 2010 Chilean earthquake [27], which also confirmed the practical performance of ESOMS from the perspective of data provision and usage. In this study, data sets obtained by the Xiaoqushan Seafloor Observatory were analyzed to extract periodicities of sea-level change and sea level anomaly (sla) was calculated based on the observed sea level data. A detailed analysis of the sea-level data on 27 February 2010 when 8.8-magnitude Chilean earthquake happened showed a peak sla residual value of 0.48 m at around 3 pm on February 28, highly coincident with the tsunami arrival time forecasted by the Pacific Tsunami Warning Center. *In situ* observatory data sets were thus expected to improve tsunami forecast models and promote the development of a tsunami warning system. With observation data continuously collected by ESOMS, long-term datasets of the Xiaoqushan Seafloor Observatory were destined to play a more important role in studying sea-land interactions of the ECS in a systematic and more detailed way.

## 5. Discussion and Conclusions

We report the progress in designing and developing a monitoring system for cabled observatories in the ECS (ESOMS), and also present a brief case study describing the use of ESOMS in the Xiaoqushan Seafloor Observatory. Major contributions are summarized as follows:

(1) A control model for an ocean observatory sensor network. The model was information oriented and was logically structured into four layers functioning in a standardized way. The model contributed to both vertical and horizontal integration of the ocean observatory sensor network, based on which different monitoring architectures and deployment strategies can be derived.
(2) A monitoring architecture for cabled observatories in the East China Sea. The architecture contained three components and enabled bidirectional information flow of observation data and control commands. The architecture contributed to a system level of plug and play mode for *in situ* sensors in the observatories, based on which components were related to model layers and designed for plug-and-play enablement.
(3) A GOE Control Method for the plug-and-play implementation of ESOMS. The method mainly actualized two processes, one of which was that the *in situ* GJB interfaced and represented every attached sensor as a Sensing Endpoint in the cabled observatory network. The other process was that the remote ESOMS utilized the same IP/Port related information modeled by OSML to create/operate a Function Node acted as agent of the *in situ* sensor. The method laid the foundation for the standardization of sensor communication and control at the system level, based on which ESOMS demanded no additional design for controlling new added sensors in the cabled observatories.
(4) A case study for the test and application of ESOMS. Experimental scenario was made for the Xiaoqushan Seafloor Observatory and a series of operational processes were tested. ESOMS presented satisfactory performance against all the functional requirements laid for the project, based on which some initial outcomes of data application were obtained.

All these contributions distinguish ESOMS from existing systems. In the context of the control model proposed in this paper, ESOMS is especially characterized in its plug-and-play enablement actualized by the GOE Control Method in the collecting and service layers. This enablement can not only vertically integrate and dynamically control sensors within a cabled observatory as conventional designs in [28,29], but also horizontally integrate a network of cabled observatories and control all the distributed sensors in a standardized and plug-and-play way. In addition, features of ESOMS include: (i) receive and store *in situ* data in a real-time way; (ii) on-line monitor the functioning of cabled observatories; (iii) remotely control the *in situ* observatory sensor network; (iv) autonomously interpret raw data for event detection and response; (v) flexibly accommodate different types of sensors with limited changes to configuration files. Although individual technologies adopted here are not necessarily new, it is the combination of the separate parts that forms the novel and robust base of ESOMS for remotely monitoring cabled observatories in a plug and play way.

As shown in the case study section, ESOMS has shown its satisfactory practical performance against all the requirements laid for the project. The successful operation of the Xiaoqushan Seafloor Observatory so far demonstrates that the architecture design and plug-and-play implementation described in this paper could be a useful reference to any new seafloor observatory network both at home and abroad. Continuous and long-term *in situ* datasets obtained in the Xiaoqushan Seafloor Observatory will in turn provide a wide range of valuable information in unprecedented temporal and spatial details to resolve scientific issues and promote research activities in the ECS.
